# Breast segmentectomy with rotation mammoplasty as an oncoplastic approach to extensive ductal carcinoma in situ

**DOI:** 10.1186/s12957-016-0825-5

**Published:** 2016-03-09

**Authors:** Bartlomiej Szynglarewicz, Adam Maciejczyk, Jozef Forgacz, Rafal Matkowski

**Affiliations:** Breast Unit, Department of Surgical Oncology, Lower Silesian Oncology Centre, Plac Hirszfelda 12, 53-413 Wroclaw, Poland; Department of Radiotherapy, Lower Silesian Oncology Centre, Wroclaw, Poland; Chair of Oncology, Wroclaw Medical University, Wroclaw, Poland

**Keywords:** Early breast cancer, Minimal-invasive breast biopsy, Core-needle biopsy, Vacuum-assisted biopsy

## Abstract

**Background:**

The aim of this study was to assess the usefulness of the breast segmentectomy with rotation mammoplasty (BSRMP) in conserving therapy for an extensive ductal carcinoma in situ (DCIS) with or without an invasive component.

**Methods:**

Thirty-six women with DCIS visible as large area of microcalcifications distributed out of the retroareolar area regardless of the quadrant were studied prospectively. All the patients underwent BSRMP and axillary procedure (31 sentinel node biopsy, 5 axillary dissection) followed by radiotherapy. In each case, follow-up was carried out carefully and special effort was made to identify postoperative complications. Cosmetic result was judged 6 months after radiotherapy by the patient herself and two surgeons being rated as poor, mediocre, medium, good or excellent.

**Results:**

Operation was completed without any difficulties in all the cases. Appropriate BSRMP was easily done after the skin marking. Regardless of the type of axillary approach, it was conveniently performed. Wound was healed by primary adhesion; skin or breast tissue necrosis did not develop. Neither haematoma nor surgical site infection was observed. In none of the patient, centralisation of the nipple-areola complex (NAC) was needed. Three patients (8.3 %) with close margins (1 mm or less) successfully underwent subsequent re-excision. The scar did not result in any impairment of arm movement. Cosmetic outcome was evaluated by the women as excellent and good in 55 (87 %) and 8 (13 %) cases, respectively, while by the surgeons as excellent, good and medium in 52 (82 %), 8 (13 %), and 3 cases (5 %), respectively.

**Conclusions:**

BSRMP is a simple and safe technique achieving good cosmetic results without NAC centralisation and giving the wide and easy access to axilla for both sentinel node biopsy and lymphadenectomy. It can be helpful in cases of extensive, radially spreading tumours (in particular DCIS or invasive cancers with intraductal component), eccentric lesions, or superficially located cancers when the neighbouring skin is excised. However, due to its limitations (long incision, difficult subsequent mastectomy, possibility of scar placement in the visible area of decollete), a careful patients’ selection should be done. Further studies are needed to assess long-term cosmetic outcomes including delayed post-radiotherapy effects.

## Background

The widespread use of breast imaging technique and adoption of screening programmes has resulted in dramatically increased incidence of ductal carcinoma in situ (DCIS). Nowadays, it represents 20–25 % of all new cases of mammographically diagnosed breast cancers [1, 2]. Microcalcifications, being its most common mammographic feature, are responsible for the detection of 85–95 % of cases of DCIS by screening mammography [2, 3].

In the era of oncoplastic surgery, more and more women with DCIS, even when it is an extensive lesion visible as large area of microcalcifications, can undergo breast preservation and achieve good cosmetic result without compromising oncological outcomes [4–7]. Because the risk of invasive cancer with nodal metastases in preoperatively diagnosed pure DCIS is very low, sentinel lymph node biopsy (SLNB) can be safely omitted [8–10]. However, in selected cases with considerable rate of underestimation, SLNB needs to be performed, preferably at the time of breast operation to avoid multistep surgery [9–11]. The optimal choice among oncoplastic techniques depends on tumour size and location as well as breast shape and volume [12]. Each oncoplastic method should obviously result in adequate local disease control and good cosmesis in regard to harmonious shape of the remodelled breast and central placement of the nipple-areola complex (NAC). On the second hand, it needs to be safe for the patient, cost-effective, and reasonable with reference to the learning curve and time consumption.

The aims of this study were to assess the usefulness of the breast segmentectomy with rotation mammoplasty (BSRMP) in conserving therapy for extensive DCIS and to evaluate early functional outcomes following this method.

## Methods

### Patients

Thirty-nine patients treated in years 2008–2014 were enrolled into the study after fulfilling the inclusion criteria: extensive DCIS (area of microcalcifications >2 cm) with or without invasive component, lack of history of ipsilateral breast cancer, location outside the retroareolar area, absence of multicentric lesions, and absence of advanced or severe breast ptosis (grades III and IV). All of them underwent minimal-invasive image-guided core-needle or vacuum-assisted biopsy before the operation to obtain histological diagnosis. Mammographic presentation included suspicious microcalcifications in all the patients, with concomitant mass or architectural distortion in 17 and 5 cases, respectively. All patients wanted to undergo breast conserving therapy. In all the cases, axillary procedure was needed to assess the nodal status because of the presence of invasive component or the important risk of underestimation of DCIS (area of microcalcifications over 4–5 cm, presence of mass, high nuclear grade with comedonecrosis). Three patients with close margins (<1 mm) had unexpectedly large DCIS with comedonecrosis and high nuclear grade on the final pathology. Because of the presence of such high-risk factors of local recurrence, we decided to perform subsequent simple mastectomy instead of the re-excision, based on the recommendations of the University of Southern California/Van Nuys Prognostic Index (USC/VNPI score 10–12). As a consequence, they were excluded. Remaining 36 women entered the analysis. SLNB was performed in 31 women (86 %). In five (14 %) patients, axillary dissection (AD) was carried out. In three patients, it was completed primarily, at the same time of breast surgery. In two others, AD was performed as a second-step procedure because of metastatic sentinel nodes. All the patients underwent conventionally fractionated (2 Gy a day) radiotherapy up to 50 Gy, with boost to the tumour site to the total dose 60 Gy administered at radiotherapist’s discretion.

For each patient, both lesion and all planned incisions were marked on the skin before surgery. Additionally, into microcalcifications or architectural distortion without mass as well as in concomitant non-palpable masses, hook-wire localisation needle was placed under the imaging guidance. In each case, intraoperative specimen radiogram was performed to confirm that all the mammographically visible abnormality was removed. In every case, an informed consent was obtained. Patients’ and lesions’ characteristics are shown in Table 1.

**Table 1:** Patient and tumour characteristics

Parameters	*n* (%)
Patient age	
Mean ± SD/median/range	54.9 ± 10.1/54.5/31–74
Family history	
Positive/negative	1 (3)/35 (97)
Menopausal status	
Pre/post	13 (36)/23 (64)
Hormone replacement therapy	
Given/not given	8 (22)/28 (78)
Patient BMI^a^	
Mean ± SD/median/range	23.7 ± 3.2/23/18–34
Tumour location—side	
Right/left	15 (42)/21 (58)
Tumour location—quadrant	
Upper outer/lower outer/lower inner/upper inner	14 (39)/10 (28)/9 (25)/3 (8)
Radiological tumour size (mm)	
Mean ± SD/median/range	37 ± 10/37/24–60
Pathological tumour size (mm)	
Mean ± SD/median/range	36 ± 9/33.5/21–53
Nuclear grade	
Low/intermediate/high	7 (19)/16 (45)/13 (36)
Comedonecrosis	
Absent/present	17 (47)/19 (53)
T stage (invasive component, *n* = 20)	
pT1/pT2	14 (70)/6 (30)
ER status	
Positive/negative^b^	31 (86)/5 (14)
Her-2 status (invasive component, *n* = 20)	
Positive/negative^c^	4 (20)/16 (80)

*n* number of patients

^a^Body mass index

^b^Positive: minimum 10 % immunostained cells

^c^Fluorescence in situ hybridisation (FISH) technique

### Surgical procedure

**Fig. 1:**
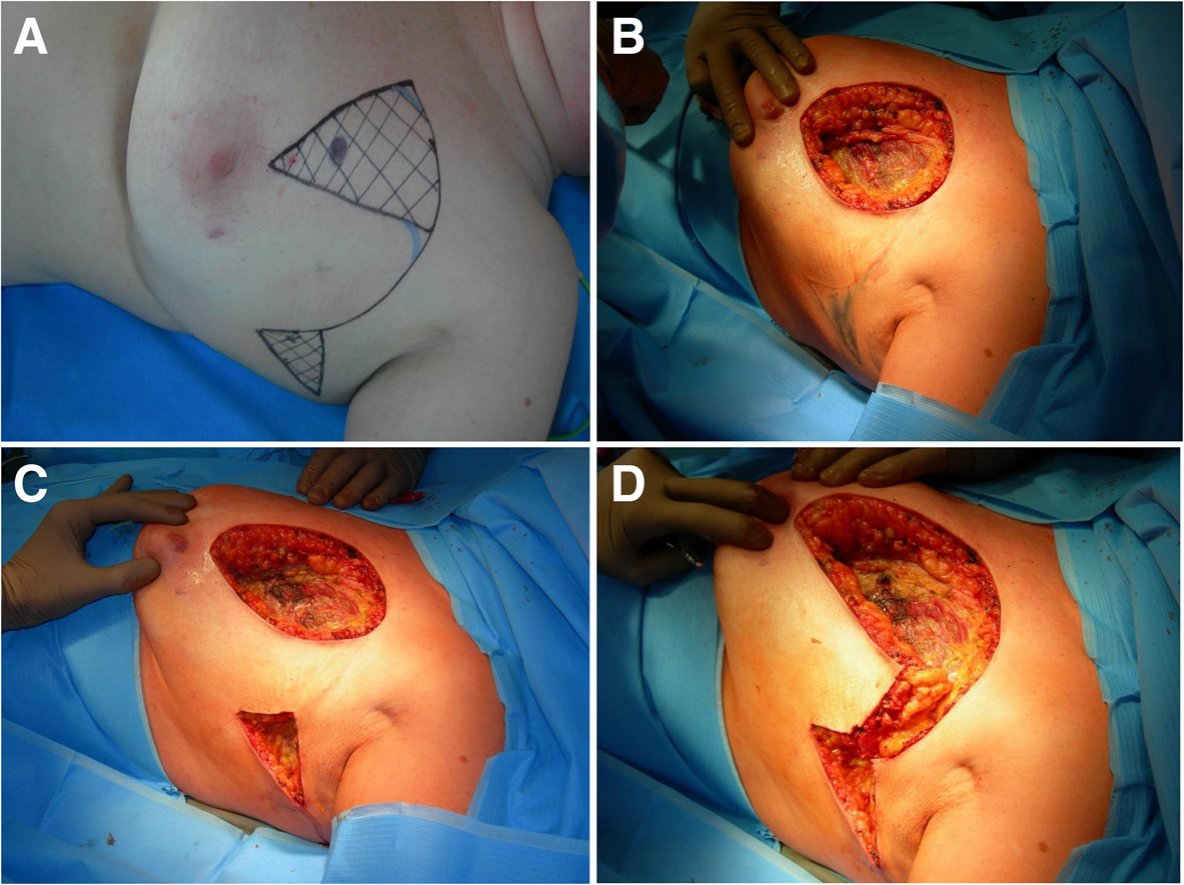
Breast segmentectomy, axillary approach, and connecting incision. **a** Skin markings. *Crossed lines*: triangles to be removed. Breast segment: extensive DCIS with invasive component. Hook-wire localisation needle inserted into invasive mass (*black oval*) under ultrasound guidance. **b** Breast segmentectomy: triangular full-thickness excision in a radial fashion. **c** Excision of axillary triangle (skin and fat tissue) at the superior area of axilla. **d** Incision connecting the bases of triangles (*upper quadrants*). Easy approach to the axillary lymph nodes

**Fig. 2:**
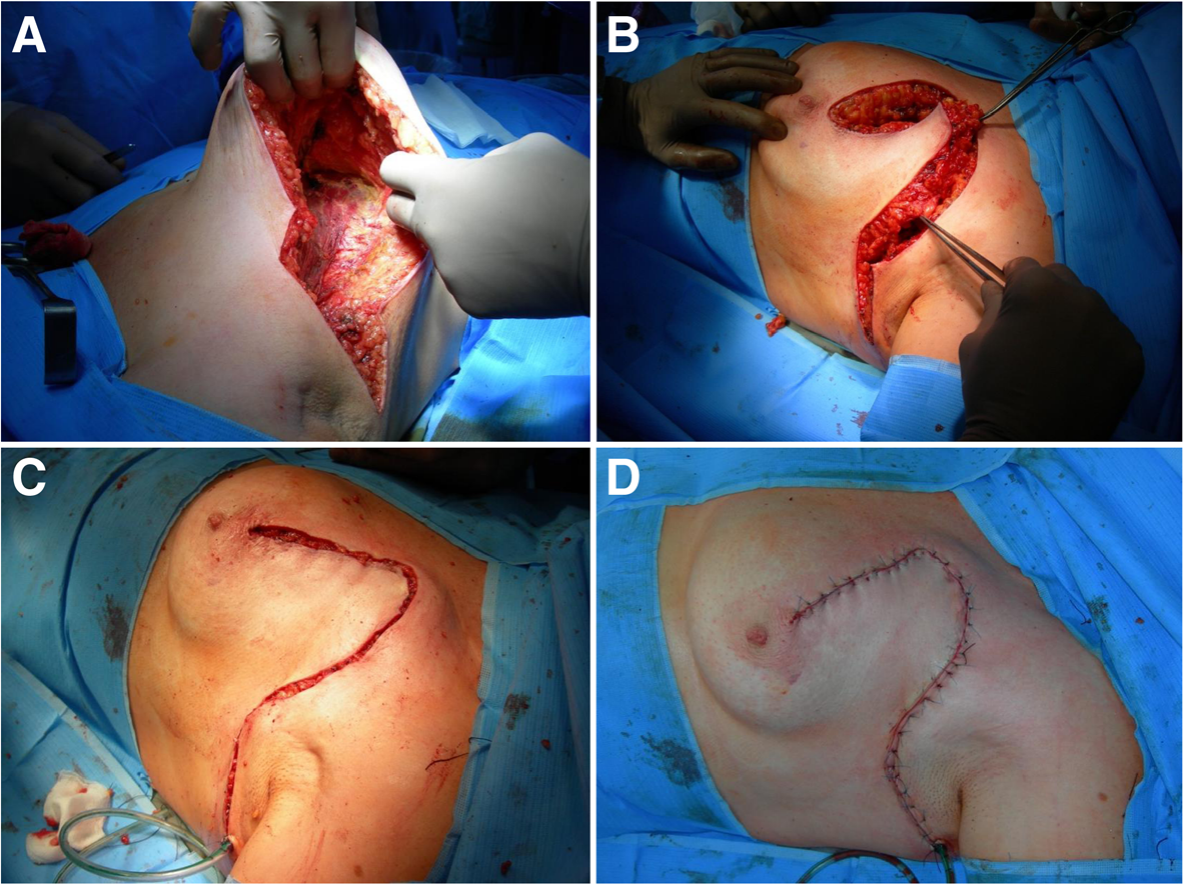
Breast rotation and mammoplasty. **a** Wide undermining of the breast glandular tissue off the pectoral fascia. **b** Breast rotation to fill the tissue defect. **c** Glandular and subcutaneous sutures. **d** Skin closing

Breast segmentectomy was done by the full-thickness excision of triangular tissue block involving skin, subcutaneous fat, and glandular tissue with the lesion and hook-wire when inserted (Fig. 1a, b). Basis of the triangle was located peripherally: in the inframammary fold for lesions in lower quadrants, at the border of glandular tissue for tumours in upper ones. Apex of the triangle was sited centrally, just at the border of NAC. When excision was performed, a special effort was made to obtain at least 1-cm macroscopic margin of normal tissue around the lesion. Tumour bed was marked with six titan clips for radiotherapy guidance. Next, the additional triangle of skin and fat tissue at the superior area of axilla was excised (Fig. 1c). Basis of this triangle was sited along the edge of pectoral muscles while the apex was located at the posterior axillary line near the edge of the latissimus dorsi muscle. It resulted in a wide access to the axillary fossa. Therefore, both SLNB and AD could be performed with ease. The following step consisted in the incision that connected the bases of the triangles (Fig. 1d). This connecting incision from the skin to the pectoral fascia was done peripherally and arranged curvilinearly. For upper-quadrant lesion, it was led along the border of breast tissue, for lower-quadrant tumours in the inframammary fold. The next manoeuvre involved a wide undermining of the breast tissue off the pectoral fascia commencing from the connecting incision, proceeding centrally to the NAC, and being continued beneath the lateral quadrants. Moreover, the skin and soft tissue overlying the axillary fossa was mobilised by the dissection along the edge of the latissimus dorsi muscle (Fig. 2a). Then, the breast tissue was lifted off the pectoral muscle and rotated medially and superiorly for upper-quadrant lesions while medially and inferiorly for lower-quadrant ones (Fig. 2b). Due to this tissue mobilisation and rotation of lateral breast parts, both defects caused by excisions of triangles were filled in (Fig. 2c, d). In two patients with metastatic sentinel nodes, a subsequent AD was performed using axillary part of previous incision without compromising the breast shape and cosmetic result of BSRMP.

All the patients had the same type of skin closure, antibiotic regimen, and drain protocol. Wound closure was done by loop sutures with Vicryl 3/0 (glandular tissue), Vicryl 4/0 (subcutaneous tissue), and Ethilon 4/0 (skin). Prophylactic antibiotics were administered at the anaesthesia induction (cefotaxime 1000 mg i.v.). Low-suction drain was placed through a separate incision before skin closure.

### Statistical analysis

Data were collected in a prospective manner. Range, mean, and median values were calculated in the term of operating time, margin width, number of removed lymph nodes, total drainage amount, number of days with drain, hospital stay, and number of postoperative office visits. In each case, follow-up was carried out carefully and special effort was made to identify postoperative complications. All complications were recorded. Cosmetic result was judged 6 months from the completion of radiotherapy by the patient herself and two surgeons being rated as poor, mediocre, medium, good, or excellent. In every case with result assessed as other than excellent, the specific cosmetic parameter influencing decreased rate was determined.

## Results

### Surgical findings and postoperative complications

Operation was completed without any difficulties in all the cases. Appropriate segmentectomy was easily done after the skin marking. Median (range) operating time and margin width were 62 min (41–115) and 11 mm (4–31), respectively. In all the patients, negative margin was obtained. In three cases (8.3 %), close margin was found (1 mm or less). It happened in small-breasted women with area of microcalcifications >4 cm (pure 5 cm DCIS in one patient, DCIS with T2 invasive cancer in two cases) and unfavourable breast/tumour size relation. Patients successfully underwent subsequent re-excision (with microscopic margin width 6, 10, and 15 mm); no more surgical attempts were needed. The median pathological size of tumour (mean ± SD, range) was 29 mm (30.6 ± 6.7, 22–47). Regardless of the type of axillary approach, it was conveniently performed. Median (range) number of removed lymph nodes during sentinel node biopsy and axillary dissection was 2 (1–4) and 18 (12–26), respectively. Median (range) total drainage amount was 80 mL (50–175) while median (range) number of days with drain was 2 (1–3). Median (range) hospital stay was 2 days (1–5). Median (range) number of postoperative office visits was 2 (1–4). Details are presented in Table 2.

**Table 2:** Operative findings and complications

Surgical findings	*n* (%)
Operating time (minutes)	
Mean ± SD/median/range	64 ± 17/62/41–115
Margin width (mm)	
Mean ± SD/median/range	12 ± 5/11/4–31
Axillary approach	
Sentinel node biopsy (SLNB)/axillary dissection (AD)	31 (86)/5 (14)
No. of removed lymph nodes (SLNB)	
Mean ± SD/median/range	2.1 ± 0.8/2/1–4
No. of removed lymph nodes (AD)	
Mean ± SD/median/range	18.6 ± 5.3/18/12–26
Total drainage amount (mL)	
Mean ± SD/median/range	89 ± 30/80/50–175
No. of days with drain	
Mean ± SD/median/range	2.2 ± 0.6/2/1–3
Hospital stay (days)	
Mean ± SD/median/range	2.5 ± 0.8/2/1–5
No. of postoperative office visits	
Mean ± SD/median/range	2.4 ± 1.3/2/1–4
Postoperative complications	
Axillary seroma/breast seroma	2 (6)/1 (3)

Wound was healed by primary adhesion; skin or breast tissue necrosis did not develop. Neither haematoma nor surgical site infection was observed. In none of the patient, centralisation of NAC was needed. The axillary part of the scar did not result in any impairment of arm movement. In none of the cases, hypertrophic scarring developed. Seroma formation occurred in three cases: in two patients in the axilla while in one woman in the breast. All patients with seroma were obese, with BMI 28, 29, and 34. Females with fluid collection in the axilla underwent AD; after SLNB, it did not develop. Seroma was effectively managed by ultrasound-guided fine-needle fluid aspiration in all these cases. Data are given in Table 2.

### Cosmetic outcomes and specific cosmetic parameters

After 6 months from the radiotherapy, cosmesis was assessed as poor or mediocre in none of the cases. Cosmetic outcome was evaluated by the women as excellent and good in 31 (86 %) and 5 (14 %) cases, respectively, while by the surgeons as excellent, good, and medium in 29 (80 %), 5 (14 %), and 2 cases (6 %), respectively. Results are presented in Table 3.

**Table 3:** Cosmetic results

Result	Women’s self-evaluation	Surgeons’ evaluation
	*n* (%)	*n* (%)
Excellent	31 (86)	29 (80)
Good	5 (14)	5 (14)
Medium	–	2 (6)
Mediocre	–	–
Poor	–	–

Three from five patients with result rated as other than excellent in self-evaluation determined a scar formation as a main reason of decreased rate. All of them had lesion sited in the superior medial quadrant, and as a consequence, a surgical scar has been formed radially and located in the area of decollete. It seems that no woman in our series accepts this scar placement. Two other women were little disappointed in the volumetric asymmetry of ipsi- and contralateral breasts. Lesions found in these patients were among the largest DCIS in the series (radiological diameters 60 and 52 mm, pathological diameters 55 and 50 mm, respectively). It resulted in a large excision and reduction of more than 25–30 % of breast volume. Despite the harmonious shape of ipsilateral breast, it has obviously given a conspicuous volumetric breasts asymmetry.

Also, in surgeons’ evaluation, the cosmetic outcome in these two women was rated as good, but not excellent. In five other patients, all the rest for whom cosmetic result was assessed as other than excellent, the reason of decreased rating was the breast shape. It was evaluated as not perfectly harmonious because of a slightly too extensive breast projection. Considering the tumour location in resected triangle, it was sited in the peripheral one-third (basis area) in 18 patients, in medium one-third (central area) in 15, and in periareolar one-third (apex area) in 3. Among patients with tumour located at the apical zone of triangle—in the periareolar one-third, this little deformation was noticed in all of them (100 %) while only in two women (13 %) with medium one-third lesion and none of the females with peripheral one-third tumour.

## Discussion

Breast-conserving surgery followed by radiation therapy has become the preferred option of locoregional treatment for the majority of patients with early-stage breast cancer. It provides equivalent survival to that of mastectomy and improves body image [12, 13]. Although standard lumpectomy use is reasonable for small cancers with favourable breast/tumour size relation, in patients with an expected volume reduction of more than 10–20 %, oncoplastic methods should be performed due a clear risk of deformity [5, 12, 13]. A breast volume reduction of more than 10 % impairs the cosmetic outcome by 50 % [14]. In contrast, oncoplastic surgery using advanced mammoplasty techniques allows resection of up to 50 % of breast volume [5, 6, 12, 13]. It results in excellent cosmesis of ipsilateral breast and also in good symmetry, when combined with immediate or delayed symmetrisation of contralateral breast [13]. On the other hand, oncoplastic surgery is associated with some disadvantages as prolongation of operative time and increased rate of local morbidity [15]. In the last decade, numerous oncoplastic techniques have been described and published. The optimal choice should be individual and based on the potential benefits balanced with possible disadvantages. All tumour-related (size, location), breast-related (ptosis, volume, NAC projection), and patient-related (age, concomitant diseases, smoking status) factors must be taken into account.

The concept of BSRMP was introduced and originally described by W. Audretsch while the technical details of this method were extensively presented during European Society of Surgical Oncology workshop at the European Breast Cancer Conference EBBC-6 (author’s session: evolution and impact of oncoplastic techniques in breast cancer treatment) [16, 17]. Since then, it has been adopted and introduced into a clinical practice in our institution. BSRMP is based on the excision in radial manner that is probably the best choice for segmentally extended cancers which account for about one third of breast malignancies [18]. This pattern of histological spread follows the ductal anatomy of the breast extending toward the NAC in a radial fashion, down the arborising ductal tree to the contiguous major lactiferous sinus at the nipple, or can extend peripherally to occupy a large portion of a breast quadrant [19].

Particularly, DCIS is generally confined to a single duct system and occupies one breast segment being distributed in a radial fashion [20, 21]. Moreover, numerous so-called multifocal DCIS are in fact contiguous disease arising within one anatomical segment in the breast, although the complicated anatomical distribution of the ducts may make it appear multicentric [22, 23]. Thomson et al. assessed and measured mammographic calcifications due to DCIS that was missed on previous mammography to obtain information concerning its growth direction and rates. Results confirm the concept of single duct system involvement by DCIS and show that its growth occurs predominantly in a radial manner: along an axis toward and away from the nipple. DCIS appears to grow in the nipple plane at equal rates toward and away from the nipple. However, it grows more than twice as fast along the axis to the nipple (5.5 mm year^−1^) as along an axis at 90° to this (2.6 mm year^−1^). Moreover, there is a significant correlation with increasing growth rates and increasing nuclear grade of DCIS. For low-, intermediate-, and high-grade DCIS growth occurred, respectively, at 1.8, 4.2, and 7.1 mm year^−1^ in the nipple plane while, respectively, at 0.2, 1.6, and 3.7 mm year^−1^ in the plane at 90° to this [24]. Authors conclude that surgical management should be based on the predictable growth pattern of DCIS, hopefully resulting in decreased rate of surgical re-interventions, i.e. re-excision of involved margins or conversion to mastectomy. Anatomical findings warrant segmental resection of DCIS and dissection along an axis drawn to the nipple (along the duct system toward and away from the nipple), as the entirety of the involved duct system is excised [23, 24].

In our series, breast resection and sentinel node biopsy or axillary dissection were both performed with ease, operative time was not long, complications were not often, and healing was uneventful. These findings show that BSRMP is feasible, easy to perform, not time-consuming, and the most important—safe for the patient. Evaluation of cosmetic outcomes suggests that good results can be obtained using this technique both if assessed by the patient herself and by the surgical team. However, the best cosmesis was achieved when breast tumour was located peripherally, in the central or basis area of resected triangle. The point is that when the tumour was sited too close to the NAC, the triangular excision was extensive to obtain clear margins. It created also the triangular tissue defect with a long triangle basis sited peripherally. When the breast tissue was lifted off the pectoral muscle and rotated to fill in the defect, the peripheral part of the breast was tightened and narrowed a little bit too much because of the too long basis of the resected triangle. It resulted in too extensive breast projection creating a kind of tubular shape rather than a perfect breast remodelling. In our opinion, the limitation of the extension of resected specimen giving harmonious breast re-modelling and excellent cosmesis in this method is approximately one eighth of breast volume in patients with moderate breast projection while about one-sixth in women with low projection. As a consequence, it does not seem to be a perfect option for patients with very high or high projection when lesion is located at the apex of the resected triangle, near the NAC. Also, women with large, pendulous breasts with grade III/IV ptosis (advanced and severe) are not the best candidates for BSRMP because they need more complex oncoplastic techniques consisting of tumour resection, breast lifting, and NAC recentralisation. Due to extensive lesions, we did not enrol patients with very small breasts (cup A). However, when small tumours are excised, BSRMP can be considered also in these cases, particularly if a wide access to axilla is needed.

None of the studied patients wanted to undergo immediate or delayed symmetrisation, including two little disappointed women. They did not recognise breast asymmetry as a significant problem impairing their self-perception or lowering the quality of life. Nevertheless, the surgical team needs to be prepared to offer such procedures. Overall, we prefer breast reduction and mastopexy using inverted-T technique with superior pedicle, because it allows to eliminate even significant asymmetry including cases with high-grade ptosis. However, in decision-making process, a breast volume, grade of asymmetry, NAC projection, and grade of ptosis should be taken into account.

To our best knowledge, just one series of BSRMP (full-thickness segmental breast resection followed by reconstruction with rotation flap advancement) has been reported. Kim et al. studied 33 patients with lower-half located breast cancer who underwent BSRMP and SLNB (64 %) or AD (36 %). All patients had negative resection margins. In none of the cases, cosmetic result 6 months after surgery was assessed as poor. Cosmesis was good (94 %) to fair (6 %) according to sum of scores of symmetry, breast shape, scarring, and NAC position [25]. Good cosmetic outcomes from this series are in concordance with our study. However, our findings indicate that also patients with lesions located peripherally in upper quadrants (particularly in upper-outer) can be successfully offered this technique.

BSRMP is associated with some disadvantages. Apart from the long incision and the possibility of scar placement in the visible area of decollete, a subsequent mastectomy can be challenging. To use traditional transverse, Stewart incision can be difficult. In our three patients excluded from the analysis because of subsequent mastectomy, it was completed using oblique incision by Orr and Y-shaped incision [26, 27]. These techniques along with the S-shaped approach seem to be reasonable options [28]. However, if the immediate reconstruction is considered, surgical scar following procedures mentioned above can be difficult to hide in the bra, impairing cosmetic outcomes.

For optimal treatment planning, numerous oncoplastic techniques should be considered and individually tailored. For central tumours, we recommend round-block approach [29], Silverstein’s batwing excision [19, 30], vertical bipedicle technique [31], or Grisotti flap [32, 33]. Lesions located more peripherally, including patients with pendulous breasts, can be removed using inferior pedicle technique (upper quadrants tumours) with hidden in the bra inverted-T scar [34] or superior pedicle technique (lower quadrants tumours) with also an inverted-T scar [13, 35] or vertical scar by Lejour and Lassus [36, 37]. Moreover, good cosmetic results in cases of lower quadrants tumours can be achieved with V-mammoplasty (lower-inner) or J-mammoplasty (lower-outer) [35] as well as with modified McKissock technique (lower inner and outer, ptotic breasts) [38] or by using a fascio-cutaneous thoracomammary flap (lower pole, small-sized breasts) [39]. However, in our opinion and based on anatomical studies and breast cancer growth pattern, BSRMP can be considered a helpful option in selected cases, such patients with extensive, radially spreading tumours (in particular DCIS or invasive cancers with intraductal component), eccentric lesions, or superficially located cancers when the neighbouring skin is excised or in women not requiring breasts lifting and NAC recentralisation.

Our report is just an observational study based on a case series. The median volume of the specimen and the tumour size/specimen volume ratio are not provided. Moreover, no comparison is done, neither to a historical cohort nor to patients treated using other oncoplastic technique. Thus, no statistically significant conclusion can be drawn. Secondly, only short-term cosmetic outcomes have been evaluated. Longer follow-up is needed to assess delayed results, including for example side effects of radiation therapy.

## Conclusions

BSRMP seems to be an interesting surgical option for oncoplastic operation of non-centrally located breast cancers with regional distribution, in particular large DCIS or invasive cancers with an extensive intraductal component. This safe and simple technique can also be considered when the breast projection is low or moderate, recentralisation of NAC is not needed, and axillary procedure is planned to be performed at the same time. However, due to its disadvantages as long incision, difficult subsequent mastectomy, or possibility of scar placement in the visible area of decollete, a careful patients selection should be done.
